# A Case of Osseous Metaplasia in a Gastric Hyperplastic Polyp

**DOI:** 10.14309/crj.0000000000000667

**Published:** 2021-09-30

**Authors:** Yingtao Zhang, Mark Friedman, Yukihiro Nakanishi

**Affiliations:** 1 Department of Pathology, Moffitt Cancer Center, Tampa, FL; 2 Department of Gastrointestinal Oncology, Moffitt Cancer Center, Tampa, FL

## CASE REPORT

**Figure 1.**
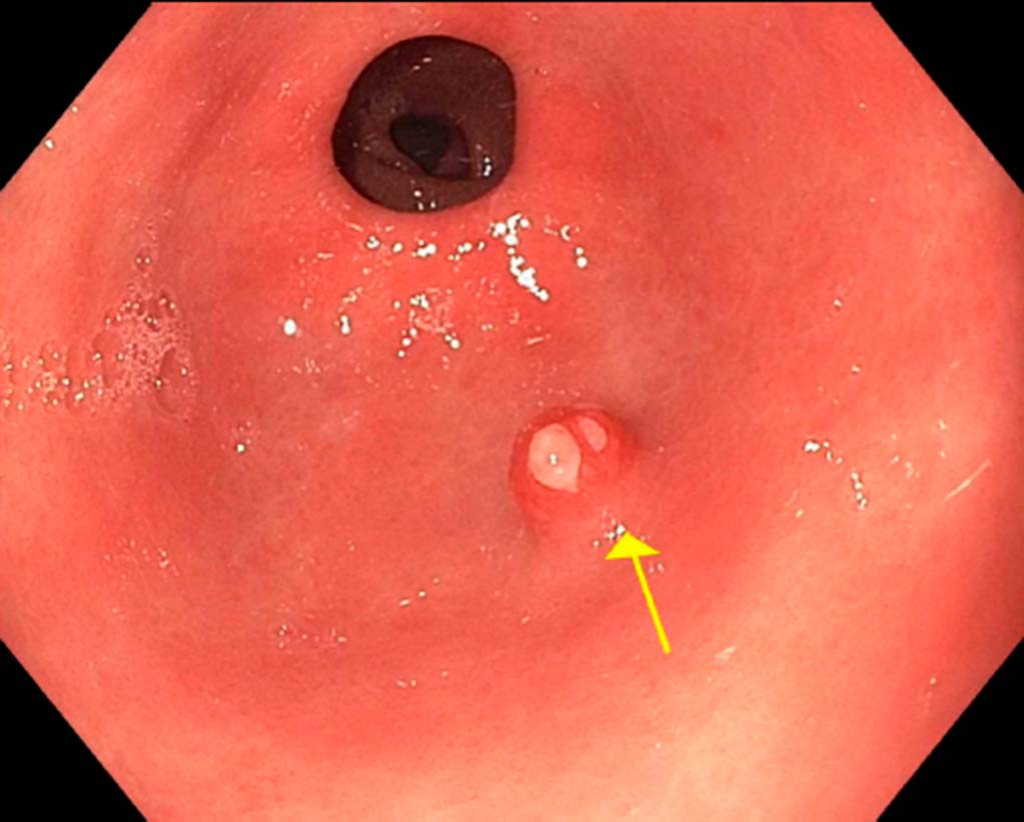
A 7-mm sessile polyp (arrow) in the antrum with reddish discoloration and erosive changes on the surface.

**Figure 2.**
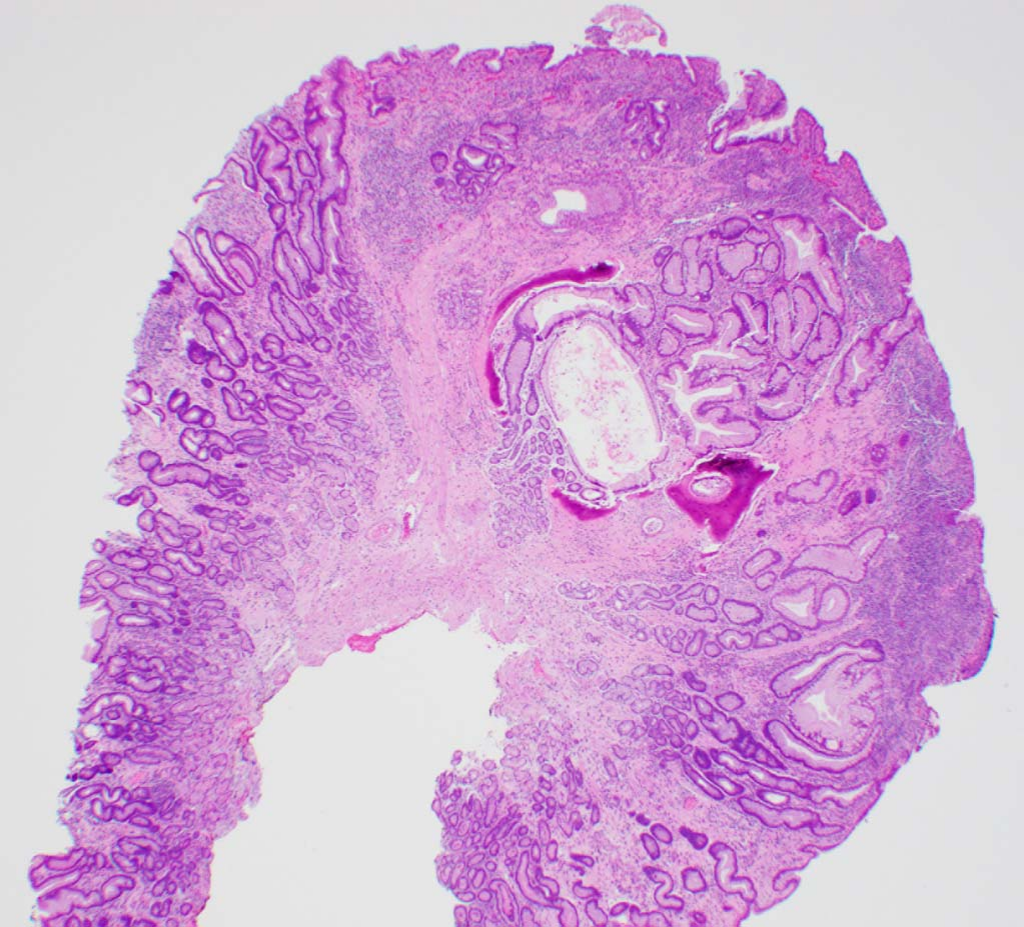
Sections of the polyp showed foveolar hyperplasia, erosion, acute and chronic inflammation, and focal granulation tissue formation.

**Figure 3.**
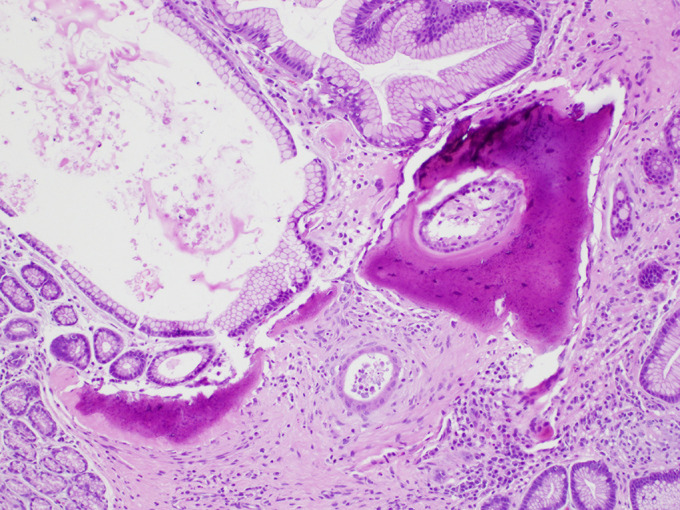
Multiple foci of woven bone formation without bone marrow surrounding dilated gastric foveolae were identified.

A 63-year-old woman with a history of hypertension, sick sinus syndrome, and Hashimoto's thyroiditis was referred to our facility for evaluation of a mass in segment 8 of the liver. Abdominal computed tomography showed an enhancing mass in segment 8 measuring 5.8 cm. The liver biopsy showed a moderately differentiated adenocarcinoma, most consistent with intrahepatic cholangiocarcinoma. A screening gastrointestinal endoscopy revealed a 7-mm sessile polyp in the antrum (Figure 1). The polyp showed reddish discoloration with erosive changes on the surface and was removed with a cold snare. No other abnormalities were identified in the stomach. Sections of the polyp showed fragments of antral-type gastric mucosa with foveolar hyperplasia, erosion, acute and chronic inflammation, and focal granulation tissue formation (Figure 2). In addition, multiple foci of woven bone formation without bone marrow surrounding dilated gastric foveolae were identified (Figure 3). No *Helicobacter* infection, intestinal metaplasia, dysplasia, or malignancy was identified histologically.

Foveolar hyperplastic polyp is a common gastric polyp usually found in the antrum and is considered as reactive changes to chronic gastritis, bile reflux, alcohol, and drugs. Histological features of foveolar hyperplastic polyp include tortuous or dilated gastric foveolae with edema, acute and chronic inflammation, and smooth muscle strands extending from the muscularis mucosae.

Osseous metaplasia/heterotopic bone formation is a well-known finding reported in various neoplastic and non-neoplastic conditions. However, osseous metaplasia in foveolar hyperplastic polyps of the stomach is extremely rare. There have been only 4 previous case reports published in the English language, one of which described 2 cases in their report.^1–4^ Among these reported 5 cases, 4 patients are men and 1 patient is a woman with ages ranging from 42 to 67 years (average age: 52.8 years). Two of 5 polyps are found in the antrum, and the size of the 5 polyps ranges from 0.3 to 3.0 cm with average size of 1.6 cm. Our current case shows clinicopathologic features similar to those of the previous case reports, including the findings of small-sized polyp found incidentally in middle-aged patients with no clinical history of hypercalcemia or any other abnormalities causing heterotopic bone formation.

The pathogenesis of osseous metaplasia remains unknown. Multiple hypotheses have been formulated, including a recent theory postulating that osseous metaplasia might occur by osteoblasts which differentiate from fibroblasts secondary to inflammation, tissue damage, or substances such as bone morphogenetic proteins.^5^

## DISCLOSURES

Author contributions: Y. Zhang wrote the manuscript. M. Friedman edited the manuscript. Y. Nakanishi revised the manuscript for intellectual content and is the article guarantor.

Financial disclosure: None to report.

Informed consent was obtained for this case report.
